# The German Health Update GEDA 2014/2015-EHIS – possibilities for international and regional assessment

**DOI:** 10.17886/RKI-GBE-2018-057

**Published:** 2018-05-16

**Authors:** Elena von der Lippe, Alexander Rommel

**Affiliations:** Robert Koch Institute, Berlin, Department of Epidemiology and Health Monitoring

The most recent survey of the German Health Update (GEDA 2014/2015-EHIS) implemented the four modules of Wave 2 of the European EHIS questionnaire (European Health Interview Survey): health status, health care, health determinants and background variables on demography and socio-economic status. In addition, the survey also covered nationally relevant topics such as health literacy, stroke knowledge, subjective social status and working conditions.

Data collection took place between November 2014 and July 2015, the reference population is the German population aged over 15 with permanent residence in Germany.

Data were collected either through an online questionnaires or, if preferred by the respondents, a printed out questionnaire sent by post. The study applied two-stage cluster sampling [1]. In order to be able to carry out analyses at federal state level, the low-population states have been over-proportionally included in the sample (oversampling).

This approach enables comparisons at European level as well as at federal state level. First analyses of selected indicators for example show that obesity and smoking prevalences in Germany are relatively close to the EU average [2]. Levels of physical activity in Germany are comparatively high, whereas levels of fruit and vegetable consumption are low. Besides the differences between Germany and the EU average there are also numerous regional differences such as in the rates of self-reported medically diagnosed diabetes and chronic-obstructive pulmonary disease or the prevalence of health impairments.

GEDA’s regularly updated information enables numerous assessments, comparisons and trend analyses. Despite the large sample and oversampling of certain federal states, conducting analyses for specific subgroups remains challenging. When reporting findings segregated by federal states, the possibility to split analysis by gender, socio-economic status and other factors should be further examined.
